# The use of SGLT2 inhibitors in people with diabetes‐related foot disease: A Delphi‐based consensus study

**DOI:** 10.1111/dom.16498

**Published:** 2025-06-11

**Authors:** Patrick Highton, Ruksar Abdala, Rachel Evley, Victoria Balasubramanian, Melanie Davies, Ketan Dhatariya, Frances Game, Clare Hambling, John R. Petrie, Sam Seidu, Solomon Tesfaye, Jonathan Valabhji, David Webb, Kamlesh Khunti

**Affiliations:** ^1^ Diabetes Research Centre University of Leicester, Leicester General Hospital Leicester UK; ^2^ National Institute for Health and Care Research Applied Research Collaboration East Midlands Leicester UK; ^3^ National Institute of Health and Care Research, Research Support Service Leicester Hub and Partners University of Leicester Leicester UK; ^4^ National Institute for Health and Care Research Leicester Biomedical Research Centre Leicester UK; ^5^ Elsie Bertram Diabetes Centre Norfolk and Norwich University Hospitals NHS Foundation Trust Norfolk UK; ^6^ Norwich Medicine School University of East Anglia Norfolk UK; ^7^ Department of Research and Development University Hospitals of Derby and Burton NHS Foundation Trust Derby UK; ^8^ NHS England London UK; ^9^ Norfolk & Waveney Integrated Care Board Norwich UK; ^10^ Litcham Health Centre King's Lynn UK; ^11^ School of Health and Wellbeing, College of Medical, Veterinary and Life Sciences University of Glasgow Glasgow UK; ^12^ Diabetes Research Unit Sheffield Teaching Hospitals NHS Foundation Trust Sheffield UK; ^13^ Department of Metabolism, Digestion and Reproduction, Faculty of Medicine, Chelsea and Westminster Hospital Campus Imperial College London London UK

**Keywords:** antidiabetic drug, diabetic neuropathy, SGLT2 inhibitor, type 1 diabetes, type 2 diabetes

## Abstract

**Aims:**

To generate expert consensus‐based clinical recommendations on the use of SGLT2 inhibitors in those with diabetes and diabetes‐related foot disease (DFD).

**Materials and Methods:**

This study employed a two‐round online Delphi technique. Participants were healthcare practitioners from a range of relevant clinical backgrounds, recruited using convenience sampling. The statements for consideration were iteratively developed by study team members with expertise in managing diabetes and prescribing SGLT2 inhibitors, supported by key professional organisations and people with lived experience of DFD. Statements were ranked using a 6‐point Likert Scale from Strongly Agree to Strongly Disagree. Consensus status for each statement was based on the Average Percent of Majority Opinions for each statement.

**Results:**

Twenty‐one participants completed round 1 of the survey, with 19 completing round 2. Participants represented a diverse range of healthcare professions, including Diabetologists, General Practitioners, Nurses and Pharmacists. Of the 25 total statements, 16 reached consensus (13 in round 1 and 3 in round 2), including: agreement on prescribing SGLT2 inhibitors to people with type 2 diabetes (regardless of ulceration status) with concurrent heart failure and/or chronic kidney disease; agreement that those with a previous healed ulcer or amputation should be prescribed SGLT2 inhibitors; disagreement that SGLT2 inhibitors per se increase amputation risk; agreement that canagliflozin should be avoided in this group.

**Conclusions:**

These findings evidence the relative confidence of experienced clinicians in prescribing SGLT2 inhibitors to those with DFD, provided that they do not have a current ulcer and that canagliflozin is not prescribed.

## 1 INTRODUCTION

Diabetes‐related foot disease (DFD) is very common: approximately 58 000 people experience a new diabetes‐related foot ulcer annually in England.^1^ People with DFD are at high risk for lower‐extremity amputation (LEA) and death.^2^ Cardiovascular disease (CVD) is the leading cause of death in DFD^3^; a recent meta‐analysis displayed a 50% 5‐year mortality in those with DFD due to CVD and infection.^4^ People with DFD are also at an increased risk for adverse renal outcomes.^5^

Sodium‐glucose cotransporter 2 (SGLT2) inhibitors are novel glucose‐lowering therapies that promote urinary glucose excretion by preventing reabsorption of filtered glucose in the proximal convoluted tubules of the kidneys.^6^ However, SGLT2 inhibitors have pleiotropic benefits, including protective effects for CVD and kidney outcomes.^7^ Three of the four currently available SGLT2 inhibitors (canagliflozin, dapagliflozin and empagliflozin) have been shown to reduce the rate of adverse CVD outcomes (including heart failure, myocardial infarction, stroke, cardiovascular mortality) in type 2 diabetes,^8^, ^9^, ^10^, ^11^ whilst ertugliflozin has displayed neutral effects comparative to placebo treatment.^12^ Furthermore, numerous studies have demonstrated potential renal benefits of SGLT2 inhibitor treatment in those with diabetes, such as preservation of renal function and reduced renal‐related death.^9^, ^13^

The Canagliflozin Cardiovascular Assessment Study (CANVAS) study, which investigated the effects of treatment with canagliflozin on cardiovascular, renal and safety outcomes in those with type 2 diabetes, identified an increased risk of minor amputation in the intervention arm,^9^ which has yet to be explained. Another study (Canagliflozin and Renal Events in Diabetes with Established Nephropathy Clinical Evaluation; CREDENCE) found no significant effect on amputation rates^14^ and so it is possible that the signal identified in CANVAS was a chance finding, as highlighted by a pooled analysis of data from both CREDENCE and CANVAS trials which identified no explanation for the difference in amputation risk between the two studies.^15^ Cohort studies have identified increased amputation risk with canagliflozin,^16^ whilst studies of other SGLT2 inhibitors (empagliflozin, dapagliflozin and ertugliflozin) have demonstrated no increased LEA risk when compared to placebo in those with type 2 diabetes.^10^, ^17^, ^18^ A recent meta‐analysis also found that SGLT2 inhibitors do not increase amputation risk when compared to DPP4‐inhibitors, whilst glucagon‐like peptide‐1 receptor agonists (GLP1a) may reduce this risk.^19^

Therefore, despite the cardiorenal benefits of SGLT2 inhibitors, concerns about amputation risk in those with DFD remain, which has likely slowed or halted the conduction of randomised controlled trials (RCTs) in this population due to safety concerns, limiting the extant body of literature in this area. It has been suggested that rare adverse events identified in some studies only should not mask the overall cardiovascular and renal benefit of SGLT2 inhibitors, especially in people with type 2 diabetes at high cardiovascular risk.^20^ However, in the absence of further robust data, some confusion remains amongst healthcare professionals. There is therefore a need to utilise other methodologies to inform the design of future trials and leverage the experience of clinicians on the use of these agents in patients with DFD.

As such, the aim of this study was to use a Delphi survey technique to generate expert consensus‐based clinical recommendations on the use of SGLT2 inhibitors in those with diabetes and DFD.

## 2 MATERIALS AND METHODS

### 2.1 Study design

The rationale and protocol for the study were identified and refined by a stakeholder engagement event in which experts in the area of SGLT2 inhibitor prescription (Diabetologists, Pharmacists, General Practitioners), supported by the study team, discussed the current evidence and identified research priorities.

This observational study was conducted using a two‐round online Delphi technique.^21^ The Delphi methodology is an interactive multi‐stage process, with each stage building on the data of the previous results in order to gain consensus from the participants. This is achieved through a series of questionnaires interspersed with controlled feedback containing the aggregated results from all participants in the study cohort.^22^ This enables the researcher to gain the most reliable consensus of opinion from the panel of experts in the field.^23^

We used this method as an expert opinion‐based consensus methodology to systematically explore opinions of relevant healthcare professionals, in order to develop consensus‐based recommendations on the use of SGLT2 inhibitors in people with DFD. The study protocol was prospectively registered on Clinicaltrials.gov (NCT06000722) and was reviewed and approved by the University of Leicester Medicine and Biological Sciences Research Ethics Committee (40318).

### 2.2 Generation of statements

The statements to be considered by participants during the Delphi process were iteratively developed by members of the study team with expertise in managing diabetes and prescribing SGLT2 inhibitors, supported by input from key professional organisations relevant to the area (Association of British Clinical Diabetologists, Primary Care Diabetes Society) and people with lived experience of DFD who reviewed the statements to ensure relevance to the patient population. The final list of statements that were included in the study is included in Table 1.

**TABLE 1:** Statements and sub‐statements that were considered in the first round of the Delphi process.

Statements and sub‐statements to be considered	Consensus status
SGLT2 inhibitors should be prescribed to all patients with diabetes, regardless of whether or not they have a current or previous foot ulcer, unless they have contraindications such as type 1 diabetes or an instance of diabetic ketoacidosis in the last year	NC
2 SGLT2 inhibitors should be prescribed to all patients with type 2 diabetes and glycaemic control above target, regardless of whether or not they have a current or previous foot ulcer, unless they have contraindications such as an instance of diabetic ketoacidosis in the last year	NC
3 SGLT2 inhibitors should be prescribed to all patients with type 2 diabetes, glycaemic control above target AND atherosclerotic cardiovascular disease (ASCVD), regardless of whether or not they have a current or previous foot ulcer, unless they have contraindications such as an instance of diabetic ketoacidosis in the last year	NC
4 SGLT2 inhibitors should be prescribed to all patients with type 2 diabetes, glycaemic control above target AND heart failure regardless of whether or not they have a current or previous foot ulcer, unless they have contraindications such as an instance of diabetic ketoacidosis in the last year	Agree
5 SGLT2 inhibitors should be prescribed to all patients with type 2 diabetes, glycaemic control above target AND chronic kidney disease (CKD), regardless of whether or not they have a current or previous foot ulcer, unless they have contraindications such as an instance of diabetic ketoacidosis in the last year	Agree
6 SGLT2 inhibitors should be prescribed to all patients with type 2 diabetes, glycaemic control above target, heart failure (HF) AND chronic kidney disease (CKD), regardless of whether or not they have a current or previous foot ulcer, unless they have contraindications such as an instance of diabetic ketoacidosis in the last year	Agree
7 SGLT2 inhibitors should be prescribed to all patients with type 2 diabetes, EVEN with glycaemic control below target, BUT have heart failure regardless of whether or not they have a current or previous foot ulcer, unless they have contraindications such as an instance of diabetic ketoacidosis in the last year	Agree
8 SGLT2 inhibitors should be prescribed to all patients with type 2 diabetes, EVEN with glycaemic control below target, BUT have chronic kidney disease (CKD), regardless of whether or not they have a current or previous foot ulcer, unless they have contraindications such as an instance of diabetic ketoacidosis in the last year	Agree
9 SGLT2 inhibitors should be prescribed to all patients with type 2 diabetes, EVEN with glycaemic control below target, BUT have heart failure (HF) AND chronic kidney disease (CKD), regardless of whether or not they have a current or previous foot ulcer, unless they have contraindications such as an instance of diabetic ketoacidosis in the last year	Agree
10 Thinking about a patient with diabetes (type 1 or 2) and a current, active foot ulcer:	
a. SGLT2 inhibitors should be prescribed to this patient to reduce their risk of future cardiovascular disease (e.g. HF) and or kidney disease	NC
b. SGLT2 inhibitors should not be prescribed to this patient	NC
c. If they are already taking SGLT2 inhibitors, these should be stopped	Disagree
d. If they are already taking SGLT2 inhibitors, they should be closely monitored as this may further increase the risk of minor amputation	NC
e. This decision would depend on other factors*Free text option to specify these factors*	‐
11 Thinking about a patient with diabetes (type 1 or 2) and a previous, healed foot ulcer:	
a. SGLT2 inhibitors should be prescribed to this patient to reduce their risk of future cardiovascular disease (e.g. HF) and or kidney disease	Agree
b. SGLT2 inhibitors should not be prescribed to this patient	Disagree
c. If they are already taking SGLT2 inhibitors, these should be stopped	Disagree
d. If they are already taking SGLT2 inhibitors, they should be closely monitored as this may further increase the risk of minor amputation	NC
e. This decision would depend on other factors*Free text option to specify these factors*	‐
12 Thinking about a patient with diabetes (type 1 or 2) and a one or more diabetes‐related amputations (minor or major):	
a. Thinking about a patient with diabetes (type 1 or 2) and a one or more diabetes‐related amputations (minor or major):	Agree
b. SGLT2 inhibitors should not be prescribed to this patient	Disagree
c. If they are already taking SGLT2 inhibitors, these should be stopped	Disagree
d. If they are already taking SGLT2 inhibitors, they should be closely monitored as this may further increase the risk of minor amputation	NC
e. This decision would depend on other factors*Free text option to specify these factors*	‐
13 Patients who take any type of SGLT2 inhibitor are at an increased risk for minor amputation*Free text option to specify if there are any types that cause greater risk*	Disagree
14 Only certain types of SGLT2 inhibitors are suitable for prescription use in patients with diabetes and current/previous foot ulcer*Free text option to specify which types*	NC
15 Canagliflozin should not be prescribed to patients with current or previous foot ulceration or amputation	Agree
16 There are certain patient characteristics, in addition to those mentioned above, that would influence my decision to prescribe them SGLT2 inhibitors*Free text option to add potential characteristics*	Agree
17 *Please use this free‐text section to create your own relevant statement if you feel that there are other relevant factors to be considered. This statement will be included for ranking in the next round of surveys*	‐

*Note*: Only those that did not reach consensus in round 1 were considered again in round 2. Consensus outcomes are presented. NC = No Consensus. The option to add additional free‐text replies was only available in round 1.

### 2.3 Participants and recruitment

Participants in the Delphi study consisted of healthcare practitioners representing a range of specialties and clinical backgrounds relevant to the use of SGLT2 inhibitors and DFD, recruited from a convenience sample using the following criteria:

Inclusion:

- Participants who are trained healthcare practitioners involved in the care of patients with current or previous DFD and knowledgeable regarding SLGT2‐inhibitors

Exclusion:

- Potential participants who do not give informed consent.
- Potential participants who are not able to participate in both rounds of the Delphi process.

Potential participants were identified via searching for members of relevant professional organisations (American Diabetes Association, Primary Care Diabetes Society, Association of British Clinical Diabetologists, International Diabetes Federation, Diabetes UK) with appropriate expertise and backgrounds, and by identifying authors of relevant recently published studies in the area, or individuals with relevant expertise known to the study team. Potential participants were then either contacted directly via email or via their professional organisation. The contact email included the participant information sheet and a link to access the Delphi survey, which included a consent form that had to be completed prior to accessing the survey. Completion of this process generated an anonymous study code for each participant. During the consent process, participants were given the option to waive their anonymity in order to be named as contributors on any resulting outputs.

### 2.4 Delphi process

The survey was hosted online (Jisc Online Surveys). The link was sent to potential participants along with the participant information sheet. Following the provision of informed consent, participants were asked to provide information regarding their demographic characteristics and professional background, including specialty or area of work and number of years worked in this area. They were then asked to consider each statement/sub‐statement (listed in Table 1) and respond to each with their level of agreement on a six‐point Likert scale (Strongly disagree; Disagree; Unsure but likely disagree; Unsure but likely agree; Agree; Strongly agree). Where appropriate, and in round 1 only, there was also the option to add free text to give further context to responses. Participants were given 3 weeks (between 5/12/2023 and 26/12/2023) to complete this process with weekly reminder emails to improve response rates.

Following the completion of round 1, the responses were collated and the statements that reached consensus (described below) were identified. The survey was then amended to include only the statements that did not reach consensus and then resent to all participants who completed round 1. When considering these statements in round 2, participants could also see the anonymous breakdown of the responses from round 1, in case that influenced their response in round 2. As with round 1, participants were given 3 weeks (between 23/04/2024 and 14/05/2024) to complete round 2 and were sent reminder emails.

### 2.5 Setting consensus

This study employed an Average Percent of Majority Opinions (APMO) approach to set the required consensus level for each round.^24^ The APMO is calculated via the following formula:

$$\text{APMO}=\begin{array}{l} \frac{\text{Aggregate of Majority Agreements}+\text{Aggregate of Majority Disagreements}}{\text{Total}\;\mathrm{no}.\;\text{of opinions expressed}}\\ \times 100 \end{array}$$

In this case, only the two response options at either end of the scale were defined as agreement (Strongly agree, Agree) or disagreement (Strongly disagree, Disagree). This method pools the responses to all statements within each round, identifies the number that fell into a ‘majority’ category (i.e., over 50% of votes to that statement) regardless of whether this was majority agreement or disagreement and expresses this as a percentage of all votes cast within the round, to determine the percentage that each statement must equal or surpass to be determined as reaching consensus. The responses to each statement were then compared against the APMO for the corresponding round (by pooling the percentages of votes to either agreement or disagreement) to identify those that did or did not reach consensus.

Other study outcomes (e.g. participant demographics) have been summarised using descriptive statistics.

## 3 RESULTS

### 3.1 Participants

In the first round of this Delphi study, 100 potential participants were invited, resulting in a response rate of 21% (*n* = 21) who completed the survey. The participant characteristics are detailed in Table 2. Participants represented a diverse range of healthcare professions and medical experts with expertise in endocrinology, diabetes care, pharmacology, nursing and academia. The most common age categories were 46–55 and 56–65 (33% each). Participants were most commonly of White (43%) or Asian/Asian British (43%) ethnicity, and 76% were male. Most (67%) were Diabetologists, and 33% had between 21 and 30 years of experience. These 21 individuals were then invited to participate in the second round, of which 19 (90%) completed the round, highlighting a high rate of retention of participants between the first and second rounds.

**TABLE 2:** Participant characteristics (*n* = 21).

	Proportion of participants^a^
Age (years)	
26–35	10%
36–45	14%
46–55	33%
56–65	33%
65+	10%
Ethnicity	
White	43%
Asian or Asian British	43%
Black, African, Caribbean	5%
Mixed or multiple ethnic groups	5%
Other	5%
Gender	
Male	76%
Female	24%
Profession	
Diabetologist	67%
General Practitioner	10%
Nurse	10%
Pharmacist	10%
Other	5%
Length of service (years)	
1–5	5%
6–10	10%
11–20	24%
21–30	33%
30+	29%
Country of work	
UK	80%
United States	10%
Austria	10%

^a^ Proportions are rounded to the nearest integer, meaning in some cases the total does not equal 100% exactly.

### 3.2 APMO cut‐off thresholds

In Round 1, the APMO cut‐off was 79.59%, meaning that statements were required to meet or exceed this level of majority agreement/disagreement in order to be determined as reaching consensus. For Round 2, the cut‐off was slightly lower at 76.21%, reflecting refined targeting as participants reassessed items based on feedback from the previous round. This approach enabled a nuanced understanding of the evolving consensus whilst maintaining rigorous standards for agreement across rounds.

### 3.3 Consensus outcomes

The numerical consensus outcomes for both rounds of the study are presented in Table 3; the question numbers map to those presented in Table 1. Of the 25 statements (including sub‐statements), nine did not reach a majority consensus. Of the remaining 16 statements, 13 reached consensus after the first round and three reached consensus after the second round. No suitable statements were provided to be added to the survey in response to question 17 in Table 1.

**TABLE 3:** Numerical consensus outcomes for each statement at Round 1 and Round 2.

Question number	Round 1		Round 2		Consensus	Direction of consensus
	APMO	79.59%	APMO	76.21%		
	Agree	Disagree	Agree	Disagree		
1	53%	47%	57%	43%	No consensus	‐
2	60%	40%	60%	40%	No consensus	‐
3	79%	21%	68%	32%	No consensus	‐
4	94%	6%	‐	‐	Achieved on Round 1	Agree
5	88%	12%	‐	‐	Achieved on Round 1	Agree
6	94%	6%	‐	‐	Achieved on Round 1	Agree
7	94%	6%	‐	‐	Achieved on Round 1	Agree
8	94%	6%	‐	‐	Achieved on Round 1	Agree
9	94%	6%	‐	‐	Achieved on Round 1	Agree
10a	53%	47%	60%	40%	No consensus	‐
10b	43%	57%	45%	55%	No consensus	‐
10c	19%	81%	‐	‐	Achieved on Round 1	Disagree
10d	79%	21%	71%	28%	No consensus	‐
11a	75%	25%	94%	6%	Achieved on Round 2	Agree
11b	24%	76%	13%	88%	Achieved on Round 2	Disagree
11c	0%	100%	‐	‐	Achieved on Round 1	Disagree
11d	54%	46%	27%	73%	No consensus	‐
12a	88%	13%	‐	‐	Achieved on Round 1	Agree
12b	13%	88%	‐	‐	Achieved on Round 1	Disagree
12c	0%	100%	‐	‐	Achieved on Round 1	Disagree
12d	67%	33%	30%	70%	No consensus	‐
13	7%	93%	‐	‐	Achieved on Round 1	Disagree
14	58%	42%	57%	43%	No consensus	‐
15	85%	15%	‐	‐	Achieved on Round 1	Agree
16	76%	24%	94%	6%	Achieved on Round 2	Agree

Consequentially, the final consensus status of each statement (Agreed, disagreed or consensus not reached) is included alongside the list of statements in Table 1. No consensus was reached regarding the prescription of SGLT2 inhibitors to all patients with diabetes (regardless of ulceration status), or similarly to patients with type 2 diabetes with glycaemic control above target with/without atherosclerotic cardiovascular disease. However, participants reached a consensus of agreement on prescribing SGLT2 inhibitors to people with type 2 diabetes (regardless of ulceration status) with concurrent heart failure and/or chronic kidney disease, regardless of glycaemic control status. Regarding patients with any type of diabetes and a current active foot ulcer, generally no consensus was reached on prescription depending on other concurrent conditions, though participants disagreed that patients already taking SGLT2 inhibitors should stop doing so. Regarding patients with a previous, healed ulcer, participants agreed that they should be prescribed SGLT2 inhibitors (or should continue taking them), though no consensus was reached on monitoring status. The same findings were observed for participants with a previous, diabetes‐related amputation. Participants also disagreed that patients taking any type of SGLT2 inhibitor are at an increased risk for amputation.

Though no consensus was reached regarding the suitability of prescribing only certain types of SGLT2 inhibitors to patients with a history of diabetes‐related ulcers, participants agreed that canagliflozin should not be prescribed to patients with current or previous foot ulceration or amputation. Participants also agreed that other patient characteristics, in addition to those described above, would influence their decision whether or not to prescribe SGLT2 inhibitors to this patient group, as described below.

### 3.4 Free‐text responses

Common responses (not already covered by the statements) included avoidance of prescribing SGLT2 inhibitors to those with type 1 diabetes or peripheral vascular disease. The comments also reiterated the belief that SGLT2 inhibitors do not increase amputation risk, though canagliflozin should be avoided in this patient group. When listing characteristics that would influence their decision to prescribe SGLT2 inhibitors to this group, participants included the following factors (not covered in the statements): heart failure (HF), CKD or renal function, risk factors, BP, weight, patient ability to understand risks and report issues, diabetes type, patient preference, current or previous infection, glycaemic control, DKA risk, pre‐conception, dietary habits, age and frailty.

## 4 DISCUSSION

Participants representing a range of clinical professions and levels of experience were largely supportive of prescribing SGLT2 inhibitors in this population, though no consensus was reached regarding patients with active ulcers. Participants supported the use of SGLT2 inhibitors to reduce CVD risk and did not support the concept of increased risk of amputation, though they agreed that canagliflozin should not be prescribed to those with previous ulceration or amputation.

The support for prescription of SGLT2 inhibitors in those with CKD and/or heart failure highlights awareness of evidence supporting the cardiorenal benefits of these medications.^25^ SGLT2 inhibitors have been shown to prevent worsening of heart failure and kidney disease and reduce mortality. The same relationship has not been observed for atherosclerotic cardiovascular disease^26^; no consensus was reached on prescription for this purpose.

The lack of consensus for active ulceration reflects uncertainty regarding safety, highlighting the need for targeted clinical trials to address this knowledge gap. Current NICE guidelines (as of October 2024) advise caution in prescribing SGLT2 inhibitors to those with a history of foot ulcer, peripheral arterial disease or lower limb amputation.^27^ This was not entirely reflected in the results of this study; participants showed more confidence in prescribing SGLT2 inhibitors in those with a history of ulceration or amputation, suggesting that the presence of an active ulcer may be the key influential factor. It may be that other novel agents (e.g., DPP4‐inhibitors) are more appropriate for this group as they have been shown to potentially improve ulcer healing.^28^

Participants overall did not believe that SGLT2 inhibitors increase amputation risk. This is supported by recent research; a nationwide study found that SGLT2 inhibitors significantly reduced amputation rates in patients with diabetes‐related foot disease compared to incretin‐based therapies,^29^ whilst another study demonstrated lower amputation risks associated with SGLT2 inhibitors compared to DPP‐4 inhibitors across various patient cohorts.^30^ In a recent review, no statistically significant increase in risk of LEA was observed for people with type 2 diabetes and peripheral arterial disease prescribed SGLT2 inhibitors compared to GLP‐1RAs. Risk of LEA also appeared to be significantly lower in people treated with SGLT2 inhibitors compared to DPP‐4 inhibitors, although it was significantly higher than when compared to sulphonylureas.^31^

However, participants were in agreement that canagliflozin should be avoided in this group. This is also in line with recent evidence; a recent systematic review and network meta‐analysis identified an increased risk of amputation associated with SGLT2 inhibitors, driven by the effect associated with canagliflozin,^32^ a finding that was mirrored in another recent review.^33^ Mechanisms behind the increased risk associated with canagliflozin have not been fully elucidated,^34^ but could include volume depletion and decreased tissue perfusion leading to tissue necrosis,^35^ and modification of bilirubin levels^36^ which may impact amputation risk,^37^ though results are inconsistent.^38^ Conversely, it may be that characteristics of the previous studies indirectly led to observed increased amputation risk associated with canagliflozin treatment. For instance: diuretics are associated with an increased amputation risk, and many patients in major relevant trials (CANVAS, EMPA‐REG, DECLARE) were receiving diuretics; elevated triglycerides and smoking may drive amputation risk, but were variably reported across trials; and baseline CVD burden and microvascular complications differed significantly across major relevant trials.^34^ Regardless of the cause being direct or indirect, clearly caution exists amongst healthcare professionals when prescribing canagliflozin.

Participants also registered other factors that might influence their decision to prescribe SGLT2 inhibitors to this population, including demographic, cardiovascular and diabetes‐related risk factors. Patient preference and ability to disease self‐manage were also considered important factors. It should be noted, however, that free‐text responses do not undergo the same ranking process as the statements, and so these data may be less robust.

Strengths of this study include the diverse nature of the participants with regards to age, job role, level of experience and ethnic background, increasing the applicability of the findings to the wider population of healthcare professionals in this area. Key stakeholders, including those with lived experience, supported the generation of statements to be included in the survey in order to ensure maximum relevance to the population of interest. Limitations include the sampling method, which may have introduced selection bias. Though it was intended to recruit those with experience relevant to the research question, it is possible that those with a greater interest in SGLT2 inhibitor use in DFD would be more likely to participate in this study, potentially introducing bias. There was also a relatively low response rate from those approached to participate in round 1 (21/100), which was possibly due to time constraints of participating clinicians or survey complexities, though the majority (19/21) were retained in round 2. Conversely, it is also possible that those with the greatest knowledge of the evidence base might be more likely to respond, limiting the benefit of increasing the sample size beyond what was achieved. Furthermore, a minimum of 12 participants has been suggested to be sufficient to achieve consensus in Delphi studies.^39^ Females were under‐represented in our sample (24%) and the survey was completed in English, so the findings may not be representative of female HCPs or those from other countries and cultures. There were also no participants with podiatry or surgery expertise. Future studies should aim to recruit a more balanced sample, including greater representation of females and participants from diverse geographical regions.

In summary, the following recommendations can be derived from the findings of this study:

1. Consideration should be given to the prescription of SGLT2 inhibitors to those with type 2 diabetes, regardless of level of glycaemia and concurrent heart failure and/or chronic kidney disease, in the absence of other contraindications, in order to realise their cardiorenal protective effects.
2. Consideration should be given to the prescription of SGLT2 inhibitors to those with type 2 diabetes and a previous history of ulceration or amputation, though caution should be exercised in those with an active ulcer.
3. Prescription of canagliflozin should be avoided in those at increased risk of amputation, though other SGLT2 inhibitors should be considered for their potential cardiorenal protective effects and protective effects against amputation compared to other novel agents.
4. Patients who are currently taking an SGLT2 inhibitor should continue to do so (unless they are taking canagliflozin, in which case they should be switched to another agent) provided that they are not experiencing adverse events or severe side effects.
5. Consider patient preference and ability to manage risks and potential adverse effects when prescribing SGLT2 inhibitors to this group.

**FIGURE 1:**
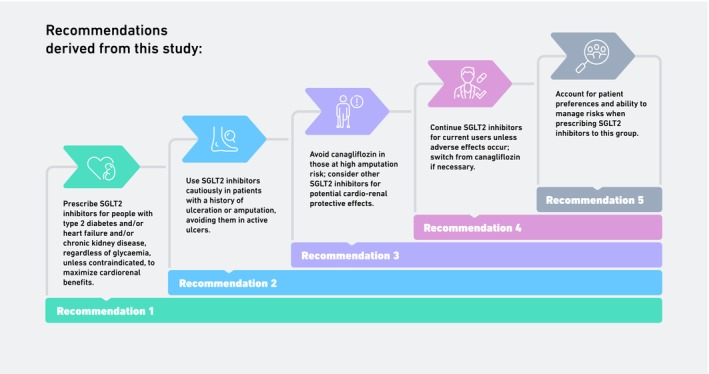
Summary of recommendations resulting from this study.

These recommendations are summarised in Figure 1.

In conclusion, the results of this Delph‐based consensus study suggest relative confidence of experienced clinicians in prescribing SGLT2 inhibitors to those with diabetes and diabetes‐related foot disease, provided that they do not have a current ulcer and that canagliflozin is not prescribed. These findings support the cautious use of SGLT2 inhibitors in type 2 diabetes patients with DFD, emphasising their benefits in heart failure and CKD whilst avoiding canagliflozin in high‐risk groups. Further data from future cardiovascular outcome trials and real‐world observational studies are required in order to explore these issues in more detail.

## AUTHOR CONTRIBUTIONS

PH designed the study, supervised recruitment, data collection and data analysis and wrote the first draft of the manuscript. RA completed data collection and data analysis. RE supported study design, data collection and analysis. VB supported data interpretation and manuscript drafting. MD, KD, FG, CH, JRP, SS, ST, JV, DW and KK conceived the study idea, supported study design, recruitment and results interpretation. All authors approved the final version of the manuscript. KK is the guarantor of this work and, as such, had full access to all the data in the study and takes responsibility for the integrity of the data and the accuracy of the data analysis.

## FUNDING INFORMATION

This project is funded by the National Institute for Health and Care Research (NIHR) under its Programme Grants for Applied Research Programme and Diabetes UK (NIHR202021). The views expressed are those of the author(s) and not necessarily those of the NIHR or the Department of Health and Social Care. The funders played no part in the design or completion of this systematic review. The project was also supported by the NIHR Applied Research Collaboration East Midlands and the NIHR Leicester Biomedical Research Centre. Support was also received from the Association of British Clinical Diabetologists and Primary Care Diabetes Society. PH is supported by an Advanced Research Fellowship award from the National Institute of Health and Care Research (NIHR303176). JV is supported by the NW London NIHR Applied Research Collaboration, the Imperial NIHR Biomedical Research Centre, and CW+ (the official charity of Chelsea and Westminster Hospital NHS Foundation Trust).

## CONFLICT OF INTEREST STATEMENT

MD has acted as a consultant/advisor and speaker for Eli Lilly, Novo Nordisk and Sanofi, has attended advisory boards for Amgen, AstraZeneca, Biomea Fusion, Carmot/Roche, Sanofi, Zealand Pharma and Regeneron, and as a speaker for AstraZeneca and Boehringer Ingelheim. She has received grants from AstraZeneca, Boehringer Ingelheim and Novo Nordisk. In the last 12 months, KD has received honoraria, travel or fees for speaking or advisory boards from: Abbott Diabetes, AstraZeneca, Boehringer Ingelheim, Eli Lilly, Menarini, Novo Nordisk, Roche and Sanofi Diabetes. JRP reports personal fees (via his employing institution) from Merck KGaA (Lectures/ Grant), Novo Nordisk (Lectures/ Advisory) Sanofi (Advisory) and IQVIA (for Boehringer Ingelheim Adjudication Committees)‐all outside the submitted work. JV was National Clinical Director for Diabetes and Obesity at NHS England from 2013 to 2023 and is now National Clinical Lead for Long‐Term Conditions at NHS England. JV is supported by the NW London NIHR Applied Research Collaboration and the Imperial NIHR Biomedical Research Centre. KK has acted as a consultant, speaker or received grants for investigator‐initiated studies for Astra Zeneca, Bayer, Novartis, Novo Nordisk, Sanofi‐Aventis, Lilly and Merck Sharp & Dohme, Boehringer Ingelheim, Oramed Pharmaceuticals, Roche and Applied Therapeutics. This article has not been previously presented elsewhere, in any format.

## PEER REVIEW

The peer review history for this article is available at https://www.webofscience.com/api/gateway/wos/peer-review/10.1111/dom.16498.

## Data Availability

The data that support the findings of this study are available from the corresponding author upon reasonable request.
